# The association between dietary mineral intake and the risk of preeclampsia in Chinese pregnant women: a matched case–control study

**DOI:** 10.1038/s41598-023-43481-4

**Published:** 2023-09-26

**Authors:** Yanhua Liu, Xinyi Wang, Wenjun Fu, Yuan Cao, Weifeng Dou, Dandan Duan, Xianlan Zhao, Shunping Ma, Quanjun Lyu

**Affiliations:** 1 Department of Nutrition, The First Affiliated Hospital of Zhengzhou University, Zhengzhou, 450052 Henan China; 2 Department of Nutrition and Food Hygiene, College of Public Health, Zhengzhou University, Zhengzhou, 450000 Henan China; 3 Department of Clinical Nutrition, The Fifth Affiliated Hospital of Zhengzhou University, Zhengzhou, 450052 Henan China; 4 Department of Obstetrics, The First Affiliated Hospital of Zhengzhou University, Zhengzhou, 450052 Henan China; 5 The Third Affiliated Hospital of Zhengzhou University, Zhengzhou, 450052 Henan China; 6 Zhengzhou Shuqing Medical College, Zhengzhou, 450064 Henan China; 7 Department of Clinical Nutrition, Luoyang New Area People’s Hospital, Luoyang, 471023 Henan China

**Keywords:** Cardiovascular diseases, Nutrition disorders

## Abstract

Previous studies on the relationship between dietary minerals and preeclampsia (PE) have given inconsistent results. The aim of this study was to further clarify the relationship between dietary minerals intake and PE in Chinese pregnant women. In this study, 440 pairs of hospital–based preeclamptic and healthy women were matched 1:1. Dietary intake was obtained through a 78–item semi–quantitative food frequency questionnaire. Multivariate conditional logistic regression was used to estimate the odds ratios (ORs) and 95% confidence intervals (CIs). Restricted cubic splines were plotted to evaluate the dose–response relationship between dietary minerals intake and PE. This study found significant inverse associations for dietary intake of calcium, magnesium, phosphorus, iron, copper, manganese and zinc and the risk of PE in both univariate and multivariate models (all *P-* trend < 0.05). After adjusting for possible confounders, compared with the lowest quartile, the odds ratio of the highest quartile was 0.74 (95% CI 0.56–0.98) for calcium, 0.63 (95% CI 0.42–0.93) for magnesium, 0.45 (95% CI 0.31–0.65) for phosphorus, 0.44 (95% CI 0.30–0.65) for iron, 0.72 (95% CI 0.53–0.97) for copper, 0.66 (95% CI 0.48–0.91) for manganese and 0.38 (95% CI 0.25–0.57) for zinc. In addition, a reverse J–shaped relationship between dietary minerals intake and PE risk was observed (*P*–overall association < 0.05). In Chinese pregnant women, a higher intake of dietary minerals, including calcium, magnesium, phosphorus, copper, iron, manganese, and zinc was associated with a lower odds of PE.

## Introduction

Preeclampsia (PE) is new–onset hypertension that develops after 20 weeks of gestation. It is characterized by proteinuria, and can progress to multiple organ dysfunction, including liver, kidney, and brain disease, and is a pregnancy–specific disease^1^. The prevalence of PE is 3–5% globally and is estimated to cause at least 42,000 maternal deaths each year^2^. In China, the incidence of PE is approximately 2.3%^3^. PE can cause irreversible short–term and long–term effects on both the foetus and the mother^4^. However, there is currently no cure for PE other than childbirth^5^. Therefore, identifying the possible causes of PE and preventing its occurrence are crucial.

Minerals, such as calcium, magnesium and phosphorus, are essential trace elements for proper body functioning. These elements are involved in many processes, including cellular metabolism, antioxidant and anti–inflammatory defense mechanisms, and also affect enzyme activities, the regulation of gene expression, and participate in protein synthesis^6^. Previous studies have found that calcium, magnesium and phosphorus play important roles in the reduction of total cholesterol concentration and the physiological regulation of blood pressure^7–12^. In addition, many minerals act as antioxidants to maintain the normal development of the placenta^13^. A lack of these elements in pregnant women increase the risk of complications, such as high blood pressure and PE^14^. In addition, inflammation has deleterious effects on placental function in humans and rodents, providing a potential mechanism underlying the development of PE^15,16^. Therefore, moderate mineral intake during pregnancy may play an important role in preventing PE.

There have been many studies on the relationship between mineral intake during pregnancy and the risk of PE. An unmatched case–control study in Ethiopia^17^ showed a significant association between low dietary calcium intake and low serum calcium concentrations and PE. A meta–analysis by Ma et al. on the relationship between zinc and PE showed that moderate zinc supplementation during pregnancy reduced the incidence of PE^18^. A cross–sectional study in India found that iron supplementation during pregnancy reduced the incidence of PE^19^. However, another studies found no association between dietary calcium and magnesium intake and PE^20^. In a randomized controlled study in China^21^, women were randomly assigned to one of the three treatment groups: folic acid, folic acid and iron, or folic acid, iron and 13 additional vitamins and minerals. No differences were found in the incidence of hypertension between the groups. Given these conflicting results, differences in dietary habits across countries and the severe consequences of PE, we conducted a matched case–control study to clarify the relationship between dietary mineral intake and PE in Chinese women.

## Methods

### Participants

This 1:1 matched case–control study was conducted from March 2016 to June 2019 in the First Affiliated Hospital of Zhengzhou University, China. This 1:1 matched case–control study was conducted at the First Affiliated Hospital of Zhengzhou University in China from March 2016 to June 2019. The inclusion and exclusion criteria were the same as those used in previous studies^22^. According to the Guidelines for the Diagnosis and Treatment of Hypertensive Diseases during Pregnancy (2015)^23^, the case group were singleton pregnant woman of childbearing age (≥ 18 years) after 28 weeks of gestation diagnosed with PE. Control subjects were women preparing for delivery at the same hospital who did not have hypertension or albuminuria and were matched for age (± 3 years), gestation week (± 1 week) and gestational diabetes mellitus (GDM). The exclusion criteria for subjects in both groups were as follows: (1) patients with heart disease, malignancy, hyperthyroidism, immune system diseases, chronic renal insufciency, and other endocrine system diseases; and (2) patients with epilepsy, depression and other mental or cognitive dysfunction.

The study was conducted in accordance with the Declaration of Helsinki and the research protocols were approved by the Ethics Committee of the First Affiliated Hospital of Zhengzhou University (Scientific Research No. 2016–LW–34). All participants signed an informed consent form before the collection of epidemiological data and biological specimens. All data used for analysis were anonymized.

### Sample size calculation

The sample size calculation was based on previous studies of the association between dietary minerals and PE (odds ratio [*OR*] = 0.42)^17^. Based on cross–sectional, cohort, and intervention studies on dietary mineral intake during pregnancy, we estimated that 15% of the general pregnant population has adequate dietary mineral intake^24^. The statistical power (*β*) was set at 80% and the significance level was set at 0.05. The sample size required for each group was calculated as 132.

### Data collection

The structured questionnaire was filled out by trained researchers during a face–to–face session. The questionnaire mainly interrogated the following: (1) food intake, (2) demographic characteristics, (3) anthropometric data and (4) nutritional supplement intake. A 78–item, semi–quantitative food frequency questionnaire (FFQ) was used to assess the dietary status of pregnant women in the 3 months before delivery. The following four levels of food–intake frequency were recorded for each item: daily, weekly, monthly or never. The reliability and validity of the FFQ have been demonstrated in previous studies^25^. The assessments of dietary minerals intake (mg/d) and energy intake (kcal/d) were calculated using the Chinese Food Composition Tables (2004)^26^ and the China Food Composition (2nd Edition)^27^. Only mineral intake from food was considered. Mineral intake from nutritional supplements was excluded. Demographic characteristics, such as age, occupation and family history of hypertension, diseases, menstruation, pregnancy and childbirth, were collected. Anthropometric data included height, weight, blood pressure.

### Statistical analysis

A paired Student’s *t*–test or Wilcoxon signed rank–sum test was used to compare the quantitative data between the two groups. McNemar’s test was used to compare qualitative data. Dietary mineral intake was adjusted for energy using the residual method^28^.

Based on the distribution in the control group, dietary mineral intake levels were divided into four equal parts (Q1–Q4), with Q1 as the control group. The association of dietary mineral intake with PE was analyzed using univariate and multivariate conditional logistic regression analysis. Confounding factors adjusted in the multivariable models were as follows: age, gestational week, pre–pregnancy body mass index (BMI), weight gain during pregnancy, educational level, income, parity, history of adverse pregnancy, family history of hypertension, use of multivitamin and mineral supplements, physical activity, energy intake and energy–adjusted vegetable and fruit intake. The median of each metric was entered into the model as a continuous variable for trend testing. A sensitivity analysis was also performed in which we analyzed the association of dietary mineral intake with PE after excluding pregnant women with GDM. A restricted cubic spline (RCS) plot was used to analyze the possible non–linear relationships between dietary minerals and PE, with the 20th, 50th and 80th percentiles used as knots. The RCS plot was calculated using Statistics Analysis System (Version 9.1, SAS Institution Inc., Cary, NC, USA) and RStudio. All other analyses were performed using SPSS Statistics (Version 21.0.0.0; IBM, Armonk, NY, USA). A two–tailed *P* value less than 0.05 was considered statistically significant.

## Results

### General characteristics and dietary minerals intake of participants

In this case–control study, 440 patients with PE were paired with 440 controls patients. There were no statistically significant differences in the distribution of age, gestational week, monthly income, adverse pregnancy history, leukocyte count or the use of multivitamin and mineral nutritional supplements between the case group and the control group. Compared with those in the control group, pregnant women in the case group were more likely to have the following: a higher pre–pregnancy BMI, more weight gain during pregnancy, lower educational level, a family history of hypertension, a lower parity, less daily energy intake, a higher level of physical activity, higher C-reactive protein levels, less vegetables and fruits intake (all* P* < 0.05). The control group had a higher dietary intake of all minerals compared with the PE group (all *P* < 0.05). The details are shown in Table 1.

**Table 1:** Characteristics and energy–adjusted dietary minerals intake of the participants.

	Case (n = 440)	Control (n = 440)	*P* value*
Age (years), mean (SD)	30.88 (5.03)	31.03 (4.85)	0.114
Gestational weeks, mean (SD)	34.17 (2.90)	34.24 (2.67)	0.066
GDM, N%	58 (13.2)	58 (13.2)	1.000
Prepregnancy body mass index (kg/m^2^), N%			
< 24	254 (57.72)	320 (72.73)	< 0.001^a^
24–27.9	129 (29.32)	90 (20.45)	
≥ 28	57 (12.95)	30 (6.82)	
Weight gain during pregnancy (kg), N%			
< 12.5	115 (26.14)	228 (51.82)	< 0.001^a^
12.5–20	206 (46.82)	186 (42.27)	
> 20	119 (27.05)	26 (5.91)	
Educational level, N%			
Primary school or less	44 (0.10)	18 (4.09)	0.005^a^
Secondary/high school	238 (54.09)	229 (52.05)	
College/university or above	158 (35.91)	192 (43.64)	
Income (Yuan/per month/person), N%			
≤ 2000	61 (13.86)	46 (10.45)	0.405
2001–4000	216 (49.09)	211 (47.95)	
4001–6000	78 (17.73)	82 (18.64)	
> 6000	59 (13.41)	81 (18.41)	
Parity, N%			
0 birth	185 (42.05)	135 (30.68)	0.001^a^
1 birth	180 (40.91)	211 (47.95)	
≥ 2 births	73 (16.59)	93 (21.14)	
Adverse pregnancy history, N%	255 (57.95)	272 (61.82)	0.257
Family history of hypertension, N%	166 (37.33)	83 (18.86)	< 0.001^a^
Multivitamin and mineral nutritional supplements users, N%	138 (31.4)	158 (35.9)	0.154
Physical activity (Met-h/d), mean (SD)	26.95 (3.96)	26.60 (4.48)	0.034^a^
Daily energy intake (kcal/d), median (IQR)	1747.21 (1509.88–2132.71)	1866.97 (1613.59–2208.97)	0.006^a^
Daily vegetables intake (g/d), mean (SD)^b^	297.80 (229.88–412.64)	332.65 (268.52–438.82)	< 0.001^a^
Daily fruits intake (g/d), mean (SD)^b^	278.19 (185.02–403.90)	332.42 (231.91–465.29)	< 0.001^a^
White cell count (10^9^/L), median (IQR)	9.30 (7.61, 11.10)	9.48 (7.60, 11,70)	0.081
C-reactive protein (mg/L), median (IQR)	11.25 (4.08, 37.38)	4.20 (2.11, 11.14)	0.028^a^
Calcium (mg/d), median (IQR)^b^	555.31 (451.84–665.65)	615.73 (516.59–730.04)	< 0.001^a^
Magnesium (mg/d), median (IQR)^b^	365.02 (319.86–409.21)	402.24 (360.07–453.94)	< 0.001^a^
Phosphorus (mg/d), median (IQR)^b^	971.00 (877.13–1080.59)	1085.93 (985.54–1205.81)	< 0.001^a^
Iron (mg/d), median (IQR)^b^	17.86 (16.55–19.53)	19.78 (18.38–21.68)	< 0.001^a^
Copper (mg/d), median (IQR)^b^	2.55 (1.83–4.52)	3.54 (2.21–5.63)	< 0.001^a^
Manganese (mg/d), median (IQR)^b^	5.47 (4.81–6.23)	5.88 (5.23–6.64)	< 0.001^a^
Zinc (mg/d), median (IQR)^b^	9.05 (8.22–9.96)	10.34 (9.31–11.26)	< 0.001^a^

*SD* standard deviation, *IQR* interquartile ranges.

*Continuous variables were evaluated using paired Student’s *t*–tests or Wilcoxon rank–sum tests. Categorical variables were evaluated using paired chi–square tests.

^a^*P* value < 0.05 as significance level.

^b^Intake adjusted for energy.

### Association between dietary mineral intake and the risk of developing PE

The relationship between dietary mineral intake and the risk of developing PE is shown in Table 2. Significant inverse and dose–response associations for the dietary intake of calcium, magnesium, phosphorus, iron, copper, manganese and zinc were found in the both univariate and multivariate models. After adjusting for possible confounders, compared with the lowest quartile, the OR of the highest quartile was 0.74 (95% confidence interval [CI] 0.56–0.98) for calcium, 0.63 (95% CI 0.42–0.93) for magnesium, 0.45 (95% CI 0.31–0.65) for phosphorus, 0.44 (95% CI 0.30–0.65) for iron, 0.72 (95% CI 0.53–0.97) for copper, 0.66 (95% CI 0.48–0.91) for manganese and 0.38 (95% CI 0.25–0.57) for zinc.

**Table 2:** Risk of PE during pregnancy according to quartiles of dietary minerals intake.

	Quartiles of dietary intake, energy–adjusted				
	Q1	Q2	Q3	Q4	*P*_trend_^#^
Calcium					
n (case/control)	178/110	113/110	78/110	71/110	
Median, mg/d (case/control)	426.23/463.41	566.41/566.01	665.46/668.33	820.88/833.01	
Crude OR (95% CI)	1.00	0.82(0.65–1.04)	0.67 (0.51–0.88)**	0.64 (0.48–0.84)**	< 0.001**
Adjusted OR (95% CI)^a^	1.00	0.84 (0.65–1.07)	0.75 (0.54–1.01)	0.74 (0.56–0.98)*	0.03*
Magnesium					
n (case/control)	209/110	108/110	69/110	54/110	
Median, mg/d (case/control)	316.31/334.40	377.75/380.55	420.08/427.77	492.46/501.31	
Crude OR (95% CI)	1.00	0.76 (0.60–0.95)*	0.59 (0.45–0.77)**	0.50 (0.37–0.68)**	< 0.001**
Adjusted OR (95% CI)^a^	1.00	0.79 (0.62–1.02)	0.72 (0.53–0.99)*	0.63 (0.42–0.93)*	0.009**
Phosphorus					
n (case/control)	241/110	94/110	60/110	45/110	
Median, mg/d (case/control)	888.92/920.24	1033.45/1036.76	1127.28/1141.37	1276.64/1309.66	
Crude OR (95% CI)	1.00	0.67 (0.53–0.85)**	0.51 (0.39–0.68)**	0.42 (0.31–0.58)**	< 0.001**
Adjusted OR (95% CI)^a^	1.00	0.73 (0.57–0.94)*	0.58 (0.43–0.79)**	0.45 (0.31–0.65)**	< 0.001**
Iron					
n (case/control)	254/109	90/113	55/109	41/109	
Median, mg/d (case/control)	16.79/17.34	18.97/19.05	20.69/20.68	23.04/23.27	
Crude OR (95% CI)	1.00	0.63 (0.50–0.81)**	0.48 (0.36–0.64)**	0.39 (0.28–0.54)**	< 0.001**
Adjusted OR (95% CI)^a^	1.00	0.72 (0.56–0.94)*	0.55 (0.39–0.77)**	0.44 (0.30–0.65)**	< 0.001**
Copper					
n (case/control)	183/109	104/113	83/109	70/109	
Median, mg/d (case/control)	1.72/1.93	2.78/2.78	4.46/4.61	8.70/8.25	
Crude OR (95% CI)	1.00	0.77 (0.60–0.97)*	0.69 (0.53–0.89)**	0.62 (0.47–0.82)**	< 0.001**
Adjusted OR (95% CI)^a^	1.00	0.92 (0.71–1.20)	0.78 (0.59–1.03)	0.72 (0.53–0.97)*	0.01*
Manganese					
n (case/control)	180/109	94/113	99/110	67/108	
Median, mg/d (case/control)	4.68/4.75	5.51/5.52	6.19/6.23	7.21/7.52	
Crude OR (95% CI)	1.00	0.76 (0.60–0.97)*	0.73 (0.57–0.94)*	0.62 (0.46–0.81)**	0.001**
Adjusted OR (95% CI)^a^	1.00	0.85 (0.65–1.10)	0.79 (0.61–1.03)	0.66 (0.48–0.91)*	0.02*
Zinc					
n (case/control)	255/109	108/113	40/109	37/109	
Median, mg/d (case/control)	8.37/8.74	9.75/9.81	10.75/10.71	11.90/12.36	
Crude OR (95% CI)	1.00	0.70 (0.56–0.87)**	0.38 (0.28–0.54)**	0.36 (0.26–0.51)**	< 0.001**
Adjusted OR (95% CI)^a^	1.00	0.74 (0.58–0.95)*	0.47 (0.33–0.67)**	0.38 (0.25–0.57)**	< 0.001**

*OR* odds ratios, *CI* confidence interval, *Q* quartile.

*P < 0.05; **P < 0.01.

^#^Determined by entering the median intake from each quartile as a continuous variable in the regression models.

^a^Adjusted OR from the conditional logistic model adjusted for age, gestational week, pre–pregnancy body mass index, weight gain during pregnancy, educational level, income, parity, adverse pregnancy history, family history of hypertension, physical activity, the use of multivitamin and mineral supplements, daily energy intake and energy–adjusted daily vegetable and fruit intake using the Enter Method.

We also performed sensitivity analyses to eliminate the influence of pregnant women with GDM on the results. As shown in Table 3, the previously observed associations persisted for all minerals. After adjusting for possible confounders, compared with the lowest quartile, the OR of the highest quartile was 0.70 (95% confidence interval [CI] 0.50–0.97) for calcium, 0.63 (95% CI 0.42–0.96) for magnesium, 0.44 (95% CI 0.30–0.64) for phosphorus, 0.38 (95% CI 0.25–0.60) for iron, 0.72 (95% CI 0.52–0.98) for copper, 0.66 (95% CI 0.47–0.93) for manganese and 0.33 (95% CI 0.21–0.53) for zinc.

**Table 3:** Risk of PE during pregnancy according to quartiles of dietary minerals intake by excluding participants with gestational diabetes mellitus.

	Quartiles of dietary intake energy–adjusted				
	Q1	Q2	Q3	Q4	*P*_trend_^#^
Calcium					
n (case/control)	162/95	93/96	66/96	61/95	
Median, g/d (case/control)	426.23/463.36	561.65/562.59	653.40/661.60	801.79/795.41	
Crude OR (95% CI)	1.00	0.78 (0.61–1.01)	0.65 (0.49–0.86)**	0.62 (0.46–0.83)**	< 0.001**
Adjusted OR (95% CI)^a^	1.00	0.78 (0.60–1.03)	0.73 (0.54–0.99)*	0.70 (0.50–0.97)*	0.02*
Magnesium					
n (case/control)	182/95	87/96	62/96	51/95	
Median, g/d (case/control)	319.90/336.28	376.51/379.15	415.80/423.07	487.19/491.56	
Crude OR (95% CI)	1.00	0.72 (0.56–0.93)*	0.60 (0.45–0.80)**	0.53 (0.39–0.73)**	< 0.001**
Adjusted OR (95% CI)^a^	1.00	0.77 (0.58–1.01)	0.69 (0.50–0.96)*	0.63 (0.42–0.96)*	0.009**
Phosphorus					
n (case/control)	214/95	78/96	53/96	37/95	
Median, g/d (case/control)	884.86/917.65	1029.28/1026.00	1118.16/1124.81	1238.77/1261.41	
Crude OR (95% CI)	1.00	0.65 (0.50–0.84)**	0.51 (0.38–0.69)**	0.41 (0.29–0.57)**	< 0.001**
Adjusted OR (95% CI)^a^	1.00	0.69 (0.53–0.92)**	0.59 (0.42–0.81)**	0.44 (0.30–0.64)**	< 0.001**
Iron					
n (case/control)	217/95	83/96	53/96	30/95	
Median, mg/d (case/control)	16.79/17.31	18.90/18.90	20.51/20.44	22.99/22.98	
Crude OR (95% CI)	1.00	0.66 (0.51–0.85)**	0.51 (0.38–0.69)**	0.35 (0.24–0.51)**	< 0.001**
Adjusted OR (95% CI)^a^	1.00	0.71 (0.54–0.94)*	0.53 (0.38–0.75)**	0.38 (0.25–0.60)**	< 0.001**
Copper					
n (case/control)	157/95	92/96	72/98	61/93	
Median, mg/d (case/control)	1.74/1.93	2.78/2.72	4.43/4.59	8.45/8.11	
Crude OR (95% CI)	1.00	0.79 (0.61–1.02)	0.68 (0.51–0.90)**	0.64 (0.47–0.86)**	0.001)**
Adjusted OR (95% CI)^a^	1.00	0.93 (0.70–1.24)	0.75 (0.56–1.02)	0.72 (0.52–0.98)*	0.01)*
Manganese					
n (case/control)	155/94	85/98	82/96	60/94	
Median, mg/d (case/control)	4.70/4.77	5.51/5.50	6.15/6.21	7.13/7.50	
Crude OR (95% CI)	1.00	0.75 (0.57–0.97)*	0.74 (0.57–0.97)*	0.63 (0.47–0.84)**	0.001**
Adjusted OR (95% CI)^a^	1.00	0.80 (0.60–1.06)	0.80 (0.60–1.07)	0.66 (0.47–0.93)*	0.02*
Zinc					
n (case/control)	223/91	92/102	39/97	28/92	
Median, mg/d (case/control)	8.37/8.72	9.68/9.72	10.62/10.65	11.71/12.16	
Crude OR (95% CI)	1.00	0.67 (0.52–0.85)**	0.40 (0.29–0.57)**	0.33 (0.22–0.49)**	< 0.001**
Adjusted OR (95% CI)^a^	1.00	0.72 (0.55–0.94)*	0.47 (0.33–0.68)**	0.33 (0.21–0.53)**	< 0.001**

*OR* odds ratios, *CI* confidence interval, *Q* quartile.

*P < 0.05; **P < 0.01.

^#^Determined by entering the median intake from each quartile as a continuous variable in the regression models.

^a^Adjusted OR from the conditional logistic model adjusted for age, gestational week, pre–pregnancy body mass index, weight gain during pregnancy, educational level, income, parity, adverse pregnancy history, family history of hypertension, physical activity, the use of multivitamin and mineral supplements, daily energy intake and energy–adjusted daily vegetable and fruit intake using the Enter Method.

### Potential nonlinear associations between dietary mineral intake and PE risk

**Figure 1:**
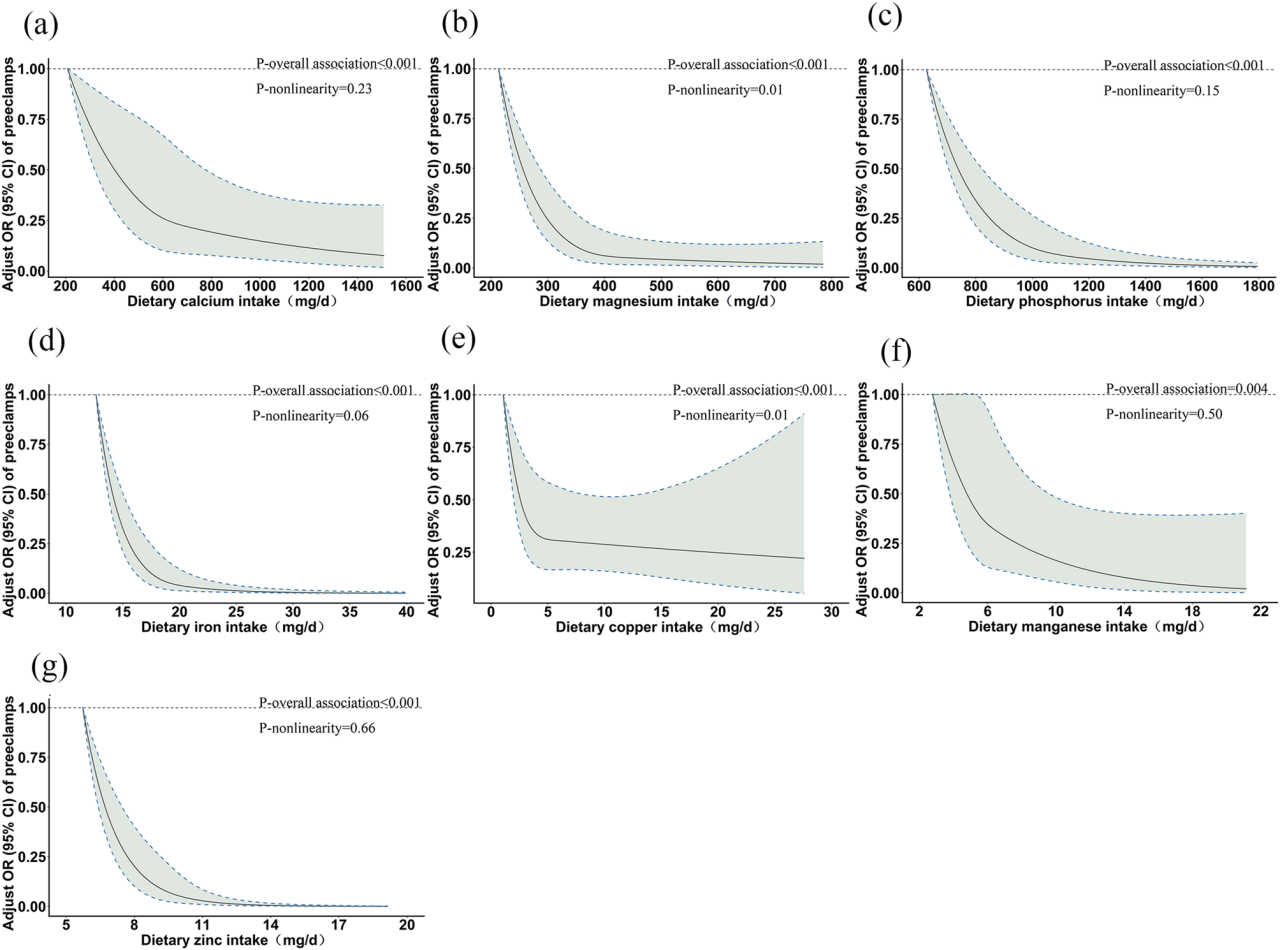
Multivariable–adjusted ORs (solid lines) and 95% CIs (dashed lines) for risk of PE according to dietary intake of calcium (**a**), magnesium (**b**), phosphorus (**c**), iron (**d**), copper (**e**), manganese (**f**) and zinc (**g**). The model was adjusted for age, gestational week, prepregnancy BMI, weight gain during pregnancy, educational level, income, parity, adverse pregnancy history, family history of hypertension, physical activity, the use of multivitamin and mineral nutritional supplements, daily energy intake and energy–adjusted daily vegetables and fruits intake. *OR* odds ratios, *CI* confidence interval.

Multivariable–adjusted RCS analyses suggested a reverse J–shaped relationship between dietary mineral intake and PE risk (Fig. 1). With increasing levels of mineral intake, the risk of PE first decreased sharply, until reaching a plateau at 670 mg/d for dietary calcium intake (*P*–overall association < 0.001, *P*–nonlinearity = 0.23), 400 mg/d for magnesium (*P*–overall association < 0.001, *P*–nonlinearity = 0.01), 1200 mg/d for phosphorus (*P*–overall association < 0.001, *P*–nonlinearity = 0.15), 25 mg/d for iron (*P*–overall association < 0.001, *P*–nonlinearity = 0.06), 5 mg/d for copper (*P*–overall association < 0.001, *P*–nonlinearity = 0.01), 6 mg/d for manganese (*P*–overall association = 0.004, *P*–nonlinearity = 0.50) and 9 mg/d for zinc (*P*–overall association < 0.001, *P*–nonlinearity = 0.66).

### Ethics approval

The study was conducted in accordance with the Declaration of Helsinki and the research protocols were approved by the Ethics Committee of the First Affiliated Hospital of Zhengzhou University (Scientific Research No. 2016–LW–34). All participants signed an informed consent form before the collection of epidemiological data and biological specimens. All data used for analysis were anonymized.

## Discussion

This 1:1 matched case–control study found an inverse association between the intake of dietary minerals, i.e., calcium, magnesium, phosphorus, copper, iron, manganese, and zinc, and the development of PE. This associations persisted after the exclusion of participants with GDM. This finding has important public health implications for the prevention of PE in pregnant women in China.

The question of whether low calcium intake is related to PE has been controversial. There are many epidemiological studies that have observed an association between calcium and the development of PE^17,29^, consistent with the results of this study (calcium intake of control group = 555.31 mg/d, case group = 615.73 mg/d, *P* < 0.001). Clinical trials have also been conducted to determine the potential benefits of preventive calcium supplementation in pregnant women^30,31^. In their meta–analysis, Patrelli et al. showed that calcium supplementation during pregnancy reduced the risk of PE more clearly in people with a low calcium intake and with a higher risk of PE than the general population^32^. However, some studies have not shown an effect of calcium on the development of PE^33–35^. Gupta et al. conducted a community–based cross–sectional study, and found no association between low calcium intake or hypocalcemia and PE (*P* = 0.57 and *P* = 0.74, respectively)^33^. In a case–control study conducted in Washington, D.C., no statistical difference in dietary calcium intake was found between a group of women with PE and a group of women without PE (*P* = 0.59)^34^. In general, the protective effect of calcium depends on the region, maternal calcium levels and health status^36,37^. More studies on the relationship between dietary calcium intake and PE are needed.

Our results show a significant negative association between dietary copper intake and PE, with RCS curves suggesting a reverse J-shaped relationship. This is consistent with the findings of Kim et al.^38^ and Bo et al.^39^. In the Fig. 1e at certain level of copper 15 mg/d on the 95% CI for the risk of PE is increased. Through reactive oxygen-mediated reactions, excess copper intake can cause DNA damage, lipid peroxidation and protein modification, thereby affecting blood pressure levels^40^. In addition, excess copper causes an increase in the amount of mature collagen and increases resistance to blood vessels^41^. However, further mechanistic studies are needed to uncover the pathways involved in this association.

Many previous studies have evaluated the associations between magnesium, phosphorus, iron, manganese and zinc and the risk of PE. Our findings are consistent with those of some previous epidemiological studies that found dietary magnesium intake to be inversely associated with the development of PE^42–44^. Jain et al. examined blood samples from pregnant women with and without PE and found that a decrease in serum magnesium concentration during pregnancy may be a cause of PE^42^. A meta–analysis of magnesium intake and PE also showed that magnesium supplementation during pregnancy reduces the risk of PE (risk ratio [RR] = 0.54, 95%CI 0.59–0.98, *P* = 0.04)^43^. Our results for phosphorus^45^, iron^46^, manganese^47^ and zinc^48^ were similar to those obtained in previous studies. Hajianfar et al. conducted a prospective cohort study to investigate the association between dietary iron intake during the first trimester of pregnancy and pregnancy outcomes^46^. The results of this study showed that higher haeme, non–haeme, and total iron intakes were associated with a lower risk of PE (haeme: crude *P* = 0.05; non–haeme iron: adjusted *P* = 0.02; total iron: adjusted *P* = 0.05). Similarly, in a study of Nigerian women, serum concentrations of zinc were significantly lower in women with PE than in women without (*P* < 0.001)^48^. These results suggest that the intake of magnesium, phosphorus, iron, manganese and zinc during pregnancy may reduce the incidence of PE.

Previous studies have suggested several mechanisms by which minerals may affect the pathogenesis of PE. Oxidative stress and inflammation have been shown to be important in the pathogenesis of adverse pregnancy outcomes^13,49^. PE is characterized by impaired placental function and may be caused by abnormal remodelling of spiral arteries. Oxidative stress due to inadequate spiral artery remodelling is an important factor associated with PE^50^. In the second trimester of pregnancy, the placenta gradually secretes a large number of anti–angiogenic factors, causing vascular inflammation, endothelial dysfunction and maternal vascular injury. The end result of altered angiogenesis is hypertension and multiple organ damage^2^. Magnesium, copper and zinc are all required for the proper functioning of enzymes like superoxide dismutase, which are needed to scavenge free radicals. A lack of these elements during pregnancy may impair the antioxidant potential of cells by reducing superoxide dismutase activity and increasing lipid peroxidation, leading to elevated blood pressure^51–53^. In addition, low levels of manganese may reduce the activity of manganese superoxide dismutase, an antioxidant located in the mitochondria, eventually leading to the accumulation of reactive oxygen species that promote the development of PE^54^. Mineral deficiencies may increase blood pressure by promoting the production of certain hormones. Iron deficiency may lead to hypoxia, which stimulates the secretion of stress hormones, e.g., norepinephrine and cortisol, which increase the risk of placental oxidative stress^55^. Low calcium intake stimulates the release of parathyroid hormone or renin, which increases intracellular calcium concentration in vascular smooth muscle cells, leading to vasoconstriction, which may cause hypertension^56^. Moderate mineral intake during pregnancy may, therefore effectively reduce the risk of PE.

This study has some limitations. First, as it is a case–control study, it was not able to discern causality. High–quality, larger randomized controlled trials are needed to evaluate the effects of multiple–mineral intakes, from both the diet and supplements, on the development of PE. Second, the dietary data collected from participants, 3 months before delivery may be subject to recall bias. To minimize the impact of recall bias on the results, face–to–face interviews were conducted by trained researchers and food photographs were used to help participants recall their dietary habits. Third, the serum concentrations of these minerals were not measured. However, a study of Iranian women, found a consistent relationship between serum and dietary zinc concentrations (*P* = 0.018)^57^. We, therefore, investigated dietary mineral intake using the FFQ, rather than the invasive and costly method of blood testing. In addition, biochemical markers such as hormones that control blood pressure were not detected. At last, despite our efforts to adjust for the influence of confounding factors, we cannot exclude the potential influence of other potential factors on our results.

## Conclusion

The findings of this study suggests that a higher intake of dietary minerals (i.e., calcium, magnesium, phosphorus, copper, iron, manganese and zinc) during pregnancy is associated with a lower odds of developing PE. In the future, larger prospective studies and biological specimens are needed to confirm these results.

## Data Availability

The datasets used and/or analyzed during the current study are available from the corresponding author on reasonable request.
