# Author Correction: The association between dietary mineral intake and the risk of preeclampsia in Chinese pregnant women: a matched case–control study

**DOI:** 10.1038/s41598-023-47163-z

**Published:** 2023-11-21

**Authors:** Yanhua Liu, Xinyi Wang, Wenjun Fu, Yuan Cao, Weifeng Dou, Dandan Duan, Xianlan Zhao, Shunping Ma, Quanjun Lyu

**Affiliations:** 1 Department of Nutrition, The First Affiliated Hospital of Zhengzhou University, Zhengzhou, 450052 Henan China; 2 Department of Nutrition and Food Hygiene, College of Public Health, Zhengzhou University, Zhengzhou, 450000 Henan China; 3 Department of Clinical Nutrition, The Fifth Affiliated Hospital of Zhengzhou University, Zhengzhou, 450052 Henan China; 4 Department of Obstetrics, The First Affiliated Hospital of Zhengzhou University, Zhengzhou, 450052 Henan China; 5 The Third Affiliated Hospital of Zhengzhou University, Zhengzhou, 450052 Henan China; 6 Zhengzhou Shuqing Medical College, Zhengzhou, 450064 Henan China; 7 Department of Clinical Nutrition, Luoyang New Area People’s Hospital, Luoyang, 471023 Henan China

Correction to: *Scientific Reports* 10.1038/s41598-023-43481-4, published online 26 September 2023

The original version of this Article contained errors. Weifeng Dou and Quanjun Lyu were incorrectly affiliated with ‘Department of Clinical Nutrition, The Fifth Affiliated Hospital of Zhengzhou University, Zhengzhou, 450052, Henan, China’. In addition, an affiliation was omitted for Xinyi Wang, Weifeng Dou, and Quanjun Lyu.

The correct affiliations for these authors are listed below.

**Xinyi Wang**

^2^Department of Nutrition and Food Hygiene, College of Public Health, Zhengzhou University, Zhengzhou, 450000, Henan, China

^3^Department of Clinical Nutrition, The Fifth Affiliated Hospital of Zhengzhou University, Zhengzhou, 450052, Henan, China

**Weifeng Dou**

^2^Department of Nutrition and Food Hygiene, College of Public Health, Zhengzhou University, Zhengzhou, 450000, Henan, China

^6^Zhengzhou Shuqing Medical College, Zhengzhou, 450064, Henan, China

**Quanjun Lyu**

^1^Department of Nutrition, The First Affiliated Hospital of Zhengzhou University, Zhengzhou, 450052, Henan, China

^2^Department of Nutrition and Food Hygiene, College of Public Health, Zhengzhou University, Zhengzhou, 450000, Henan, China

The original Article has been corrected.

